# Large carnivores and naturalness affect forest recreational value

**DOI:** 10.1038/s41598-022-17862-0

**Published:** 2022-08-11

**Authors:** Marek Giergiczny, Jon E. Swenson, Andreas Zedrosser, Nuria Selva

**Affiliations:** 1 Faculty of Economic Science, University of Warsaw, ul Długa 44/50 00-241, Warsaw, Poland; 2 Faculty of Environmental Sciences and Natural Resource Management, Norwegian University of Life Sciences, Box 5003, 1432 Ås, Norway; 3 Department of Natural Sciences and Environmental Health, University of South-Eastern Norway, 3800 Bø, Telemark Norway; 4 Institute for Wildlife Biology and Game Management, University for Natural Resources and Life Sciences, 1180 Vienna, Austria; 5 Institute of Nature Conservation Polish Academy of Sciences, 31-120 Kraków, Poland

**Keywords:** Ecology, Environmental sciences, Environmental social sciences

## Abstract

Recreation is a crucial contribution of nature to people, relevant for forest ecosystems. Large carnivores (LCs) are important components of forests, however, their contribution to forest recreational value has not yet been evaluated. Given the current expansion of LC populations, the ongoing forest conservation debate, and the increasing use of nature for recreational purposes, this is a timely study. We used discrete choice experiments and willingness-to-travel to determine people’ preferences for both forest structural characteristics and presence of four LC species in Poland (N = 1097 respondents) and Norway (N = 1005). In both countries, two-thirds of the respondents (termed ‘wildness-positive’) perceived LCs as contributing positively to forest recreational value and preferred to visit old forests with trees of different species and ages and presence of dead wood (i.e. natural forests). Respondents with negative preferences towards LCs preferred more intensively managed forest (‘wildness-negative’); their preferences were stronger than in wildness-positive respondents and in Norway. Preferences towards wild nature were highly polarized and there were hardly neutral people. Our results showed a strong link between preferences for LC presence and forest structure, and reflected the dualism of human-nature relationships. This study highlights the need to consider the contribution of forests and LCs to human recreation services in ecosystem management policies.

## Introduction

The idea of wildness as an escape from the stranglehold of civilization was developed by nineteenth century romantics, such as John Muir and Henry David Thoreau, and has since become embedded in conservation policies^1,2^. Defined as a function of both naturalness and freedom from human control^3^, the degree of wildness has declined at an alarming rate, particularly in the last decades, and its preservation and restoration has been an important focus of conservation efforts^4,5^. The restoration of wildness is the main goal of rewilding (the restoration of an area to its natural uncultivated state), an important topic in conservation that is currently under intense debate^6,7^. Whereas the ecological aspects of rewilding, such as restoration of ecosystem processes, ecological integrity, landscape connectivity, reintroduction or increase of large predator populations, natural disturbances, or trophic complexity have been widely debated^6–9^, the societal aspects have received comparatively less attention. Recently, the importance of people’s perceptions and experiences of wildness, as well as the benefits and contributions to people from restoring nature, have been highlighted as crucial for the success of rewilding initiatives^7^.

The feeling of wildness may represent an important nonmaterial contribution of nature to people. Nonmaterial contributions are defined as nature’s effects on subjective or psychological aspects supporting people’s quality of life, at the individual or collective level^10^. They are an important part of cultural ecosystem services^11^. A growing body of evidence supports that nature and wildness experiences contribute to human health and well-being [e.g.,^12,13^]. Immersion in wildness may improve psychological, emotional and social health^14^ and can promote human resilience and flourishing even in urban environments^15^. As the human impact on Earth’s ecosystems continues to grow^16^, people’s need for wildness experiences may become increasingly relevant. In this context, it is important to understand human-nature relationships in an increasingly crowded and transformed world.

Forest ecosystems, particularly natural or old-growth forests, have exceptional environmental values that are widely recognised in conservation and have been linked to wildness [e.g.^4,17^]. The amenity values of forests, such as scenic beauty and recreation, are also becoming increasingly relevant and the public demand for recreational services of forests is on the rise^12,18^. Unmanaged forests or forests managed for biodiversity are more attractive to people^18^. Only 0.7% of Europe’s forest area is classified as primary forest^19^, indicating that most European forest ecosystems have been altered, particularly since the emergence of industrial forestry in the middle of the eighteenth century^20^. Large carnivores (LCs) are an essential component of ecosystems, and their restoration has been central to the definitions of rewilding^7,8^. Many people regard LCs as symbols of wild nature^21^ (2018), whereas others perceive them as problematic and even as hazards^22^. Today, LC populations are recovering and recolonizing human-dominated landscapes in Europe, mostly in mountainous and forested areas, a pattern partly associated with increases in forest cover^23,24^. Numerous studies have evaluated public attitudes and perceptions towards LCs [e.g.^25,26^], or assessed the recreational value of forests based on forest structural attributes^18,27,28^. However, to our knowledge, no study has combined these two important aspects and considered the effect of LCs as an attribute of the recreational value of forest ecosystems.

**Figure 1:**
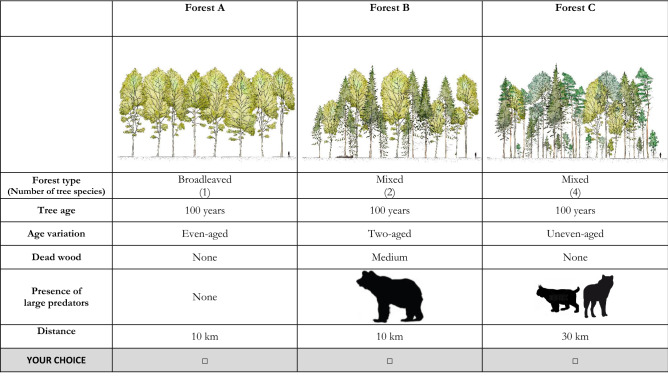
Example of card used in the choice experiment for assessing the preference for forest attributes in Poland and Norway, where the respondents had to select one single option.

In this study, we estimated the value of LCs for human recreation in forests in relation to forest characteristics. Using discrete choice experiments, we estimated ‘willingness-to-travel’, i.e., the distance people would be willing to travel on average to visit a forest with a given set of structural attributes and whether or not it was inhabited by LCs—the brown bear (*Ursus arctos*), grey wolf (*Canis lupus*), Eurasian lynx (*Lynx lynx*), and/or wolverine (*Gulo gulo*)—(see Table 1 and Fig. 1). We focused on two countries in Europe with a priori different context and social perspectives on LC and forest management, Poland and Norway (Table S1). In Poland, all LC species are strictly protected and only problem animals can be removed under a special permit; wolf and bear numbers are increasing. Norway allows regulated hunting of LCs, even though they are all red-listed, to maintain stable populations and to ensure that depredation of free-roaming sheep and domestic reindeer is kept at low levels. The abundance of LCs is comparable in both countries, except for the wolf, which is an order of magnitude more abundant in Poland, and the wolverine, which is present only in Norway [^23^, Table S1]. Forest management also differs between the two countries. In Poland, over 80% of forests are public, whereas in Norway, ~ 85% of forests are privately owned (Table S1). In both countries, most of the forest ecosystems is intensively managed for timber production, whereas other ecosystem services provided by forests, such as recreation, are often ignored.

**Table 1:** Attributes and levels used in choice experiments to investigate public forest preferences in Poland and Norway.

Forest attribute	Description	Levels [base level]
Forest age	The average age of the upper tree storey in a forest. Respondents were informed that the height of the tree canopy is related to the age of the stand, respectively: 40 years—ca. 8 m. in height, 70 years—ca. 16 m, and 100 years—ca. 26 m. The figure of a person was added in the illustrations as a reference (see Fig. 1)	[Age 40]—young forest stand, of about 40 years and ca 8 m height Age 70—stands of intermediate age and ca 18 m height Age 100—old forest, with stands on 100 years on average and ca 26 m height
Forest type	Graphics of Scots pine (*Pinus sylvestris*) and Norway spruce (*Picea abies*) were employed to visualize coniferous species; oak (*Quercus robur*), birch (*Betula pendula*) and beech (*Fagus sylvatica*) to visualize broadleaved tree species. *Coniferous* forest in both countries was always composed of one species only, in half of the choice cards it was Norway spruce and Scots pine in the second half. *Mixed* forests could be composed of 2 or 4 species—2 in Norway and 2 or 4 in Poland. In the case of Norway, it was always a mixture of birch with Scots pine or Norway spruce, whereas in Poland any combination of coniferous and broad-leaved tree species was equally likely	Polish levels: [Coniferous—1 species] Broadleaved—1 or 3 species Mixed—2 or 4 species Norwegian levels: [Coniferous—1 species] Mixed—2 species
Number of tree species	This attribute is related to the attribute *Forest type.* To mimic reality, based on the tree species combinations most commonly found in both countries, a maximum of 4 tree species was used in Poland (available tree species levels: 1, 2, 3 and 4) and a maximum of 2 species in Norway (either 1 or 2 tree species)	
Variation in tree age	It reflects how diverse the stand is in relation to the age of the trees	[Even-aged]- forest composed of a single age class, typical for a forest plantation Two-aged- forest with trees of two distinct age classes Multi-aged- forest with trees of three or more distinct age classes, typical for natural forests
Dead wood	Amount of natural dead wood (standing and fallen) in a forest. Respondents were informed that this attribute refers to large pieces of natural dead wood to avoid confounding it with the presence of wood debris from harvesting and thinning	[Low]—no dead wood in the forest Medium- intermediate amount High—level similar to those in natural forest
Large carnivore presence	Each forest was described by the presence of large carnivore species: *grey wolf*, *Eurasian lynx***,** and *brown bear* in Poland and Norway, with additionally *wolverine* in Norwegian forests	Poland: Wolf, Lynx, Brown bear Norway: Wolf, Lynx, Brown bear, Wolverine
Distance	The distance from the respondent’s home to a forest the respondent would visit. Typically, cost is expressed in monetary terms in choice experiment studies, and this attribute is later used to calculate the willingness-to-pay. However, as our study has a recreational context, the cost was expressed as the additional distance a person would be willing to travel to visit a forest described by a given set of attributes	5, 10, 20, 30, 60 km- distance needed to travel in order to visit (or avoid) a forest with given attributes

The reference level for the statistical analysis is indicated in brackets.

Here we use a questionnaire survey with choice experiments to assess (1) whether and to what extent the presence of LCs contributes to the perceived recreational value of forests; (2) how visitor preferences in relation to LC presence are associated with forest structural features, and (3) if there were differences between Poland and Norway in relation to these two questions (see Table 1 and Fig. 1). We further discuss how visitors’ preferences regarding forest structural attributes and LC occurrence relates to the perception of wildness. Given the expansion of LC populations as well as the increasing demand for forest recreational use in Europe, our research provides insights into public preferences for forest recreation that can contribute to more sustainable forest and LC management policies.

## Results

The models were estimated from a representative sample of 1097 respondents in Poland and 1005 respondents in Norway. We report the descriptive statistics in Table 2. On average, Norwegians visited forests more often (32 visits annually) compared to Polish respondents (21 visits). The main purpose of the visits was ‘Walking’ (79% in Poland, 87% in Norway) and ‘Observing nature’ (51% in Poland, 62% in Norway). ‘Hunting’ was the least reported purpose of the visits in both countries, 1% in Poland and 6% in Norway (Table 2). In both countries, women and older people had a more negative attitudes to nature, while higher number of visits to the forest, particularly those aiming to observe nature, were associated to positive views (Table S2).

**Table 2:** Descriptive statistics of respondents from the general public to investigate forest preferences in Poland (N = 1097) and Norway (N = 1005) in 2016.

Respondents’ description	Poland				Norway			
	Mean	SD	Min	Max	Mean	SD	Min	Max
Age	40.60	13.40	18	70	45.31	14.86	18	70
Gender (Women)	0.52				0.51			
Number of forest visits in the last 12 months	22.59	26.35	0	100	31.52	29.83	1	100
**Main purpose of the visit**								
Walking	0.79	0.40	0	1	0.87	0.34	0	1
Observing nature	0.52	0.49	0	1	0.61	0.49	0	1
Sport	0.18	0.38	0	1	0.23	0.42	0	1
Mushroom/berry picking	0.56	0.49	0	1	0.34	0.47	0	1
Hunting	0.01	0.09	0	1	0.06	0.23	0	1

The signs and levels of all significant estimates of the Multinomial Logit model and the means of the Mixed Logit model were of similar magnitude (Table 3). This indicates that the results of our study were not sensitive to the use of either model. In the case of the Mixed Logit model, we obtained a log-likelihood improvement of 1938.31 units for Poland and 1666.63 units for Norway, which came at the cost of 15 additional parameters (i.e., the mean and SD of random parameters) for Poland and 13 for Norway. Both of these changes in the log-likelihood, based on the log-likelihood ratio test, were highly significant and indicated that there was a substantial random preference heterogeneity in both countries (see Table 3 for details). Therefore, we focused our attention on the results from the Mixed Logit model.

**Table 3:** Output of the multinomial logit model and mixed logit model assessing respondents’ preferences in relation to forest structural attributes and large carnivore presence in Poland and Norway.

Attributes	Poland							Norway						
	Multinomial logit model		Mixed logit model					Multinomial logit model		Mixed logit model				
	Mean WTT	SE	Mean WTT	SE	SD	SE	Share negative	Mean WTT	SE	Mean WTT	SE	SD	SE	Share negative
**Forest attributes**														
Broadleaved 1	− 9.62***	2.73	− 5.01***	1.83	13.01***	2.51	0.65							
Broadleaved 3	10.8***	2.21	9.62***	1.53	3.21	3.77	< 0.01							
Mixed 2	8.97***	2.71	8.54***	1.76	2.96	2.90	< 0.01	11.2***	0.97	10.83***	0.96	15.8***	1.22	0.25
Mixed 4	16.28***	2.05	14.5***	1.53	18.47***	2.18	0.22							
Age 70	23.77***	3.03	11.98***	1.93	0.04	2.32	< 0.01	7.52***	1.32	4.48***	1.16	9.55***	1.75	0.32
Age 100	29.79***	4.08	16.61***	2.68	3.65	2.68	< 0.01	13.09***	1.51	9.47***	1.22	5.63**	2.76	0.05
Two-aged	7.62***	1.47	7.17***	1.09	2.54	1.94	< 0.01	− 4.15***	1.28	0.52	1.08	3.47**	1.75	0.44
Uneven-aged	4.57**	1.98	3.32**	1.37	7.45***	1.94	0.33	− 1.82	1.28	2.24**	1.09	0.71	1.91	< 0.01
Dead wood Medium	2.12	2.21	0.64	1.55	12.72***	1.83	0.48	2.45**	1.09	2.46***	0.92	1.92	2.36	0.10
Dead wood High	5.44***	1.48	3.89***	1.17	15.57***	1.70	0.40	0.02	1.11	− 2.21**	1.09	17.9***	1.48	0.55
**Large carnivore presence**														
Bear	− 3.29**	1.39	− 5.85***	1.61	41.46***	2.47	0.56	− 4.95***	0.99	− 13.06***	2.22	39.32***	2.20	0.63
Lynx	15.25***	1.58	11.38***	1.48	32.07***	2.01	0.36	6.69***	0.97	5.34***	1.16	22.45***	1.47	0.41
Wolf	− 0.78	1.38	− 5.15***	1.54	37.1***	2.10	0.56	− 3.17***	1.00	5.04***	1.31	25.05***	1.57	0.58
Wolverine								− 1.46	0.95	− 1.1	1.14	22.18***	1.54	0.52
**Model diagnostics**														
Log-likelihood	− 10,470.82		− 8804.19					− 9482.85		− 7544.54				
Pseudo R^2	0.0504		0.2016					0.0404		0.2365				
Observations	8776							8040						
Respondents	1097							1005						

Estimates are expressed in willingness-to-travel (WTT, kms). ‘Share negative’ is the proportion of respondents who have negative willingness-to-travel.

The levels of significance are as follows: *0.1, **0.05, ***0.01.

### Preferences for forest structural characteristics

**Figure 2:**
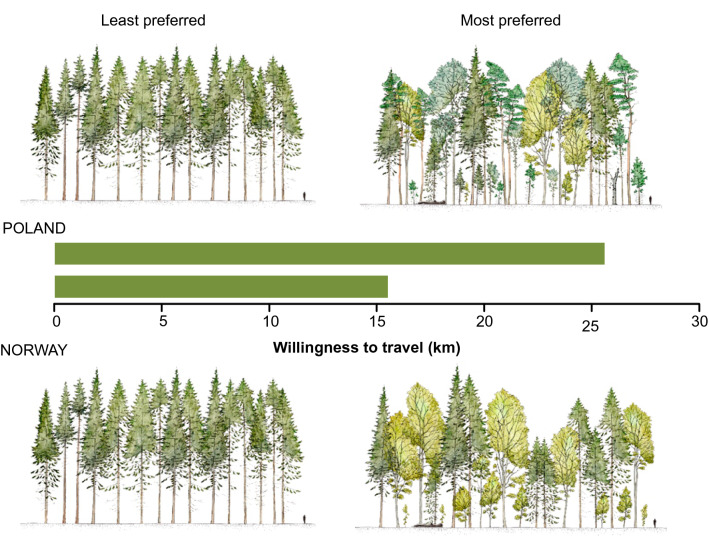
Visual representation of the least and most preferred forest structure by respondents in Poland (N = 1097) and Norway (N = 1005), expressed as willingness-to-travel (kms). The least preferred forest structure in both countries was young (40 years) even-aged coniferous monoculture without dead wood. As the age of the stand had a strong effect on the willingness-to-travel, to better reflect the differences in preferences for forest structures, the figure shows the least and most preferred forest of the same age.

In both countries, the least preferred forest to visit was of the youngest age (*Age 40*), composed of one species of coniferous trees (*Coniferous 1)* of the same age (*Even-aged)* and without *Dead wood* (Table 3, Fig. 2). In general, respondents from both countries found it more attractive to visit older forests, with different tree species and ages, and a certain amount of dead wood. There was a strong and positive relationship between *Forest age* and recreational value. In both countries, *Age 100* was the most preferred level, and *Age 70* was preferred over *Age 40* (Table 3). Respondents in Norway were willing to travel an extra 9.5 km and respondents in Poland an extra 16.6 km to visit a forest with *Age 100* compared to the reference level (*Age 40)*. The respondents in both countries preferred forests with some *Variation in tree age*, with *Multi-aged* stands being the most preferred in Norway and *Two-aged* stands being the most preferred in Poland. The willingness-to-travel values for the most preferred levels of *Variation in tree age* were 2.2 km in Poland and 7.2 km in Norway. In Poland, both levels of tree-age variation (i.e. *Two-aged* and *Multi-aged*) were preferred over the reference level (*Even-aged*), whereas in Norway the *Two-aged* level was not statistically different from the reference level.

Regarding *Forest type*, Norwegians were on average willing to travel an extra 10.8 km to visit *Mixed 2* compared to *Coniferous 1* (Table 3). In Poland, the corresponding mean value for *Mixed 2* was 8.5 km. Polish respondents had a strong and positive preference for more tree species; *Mixed 4* was strongly preferred over *Mixed 2* (i.e., willingness-to-travel of 14.5 km vs. 8.5 km) and *Broadleaved 3* was preferred over *Broadleaved 1* (i.e., 9.6 km vs. − 5.0 km). When controlling for *Forest type*, increasing the number of tree species (within the studied range) raised the forest recreational value of a given forest. Because the number of tree species in our survey varied across forest types (i.e. *Coniferous 1, Broadleaved 1, Broadleaved 3*, *Mixed 2* and *Mixed 4*), it was difficult to compare preferences for given forest types. However, the Polish respondents mostly preferred forest type *Mixed 4* (willingness-to-travel was 14.5 km); *Mixed 2* and *Mixed 4* were systematically preferred over *Coniferous 1*. *Coniferous 1* (the base level) was preferred over *Broadleaved 1* (0 km vs. − 5 km) in Poland. After controlling for the number of tree species, the preference relationship in Poland for the forest type was *Mixed* > *Coniferous* > *Broadleaved*. In Norway, the maximum number of tree species was 2, and *Mixed 2* was strongly preferred over *Coniferous 1*.

The relationship between the amount of *Dead wood* and willingness-to-travel was nonlinear in both countries. It had an inverted U-shape in Norway, with the *Dead wood Medium* level being the most preferred (2.5 km) and *Dead wood High* being statistically less preferred than the base level (*No dead wood*). In Poland, *Dead wood Medium* was not statistically different from the reference level and *Dead wood High* was the most preferred (3.9 km).

### Preferences for large carnivores

**Figure 3:**
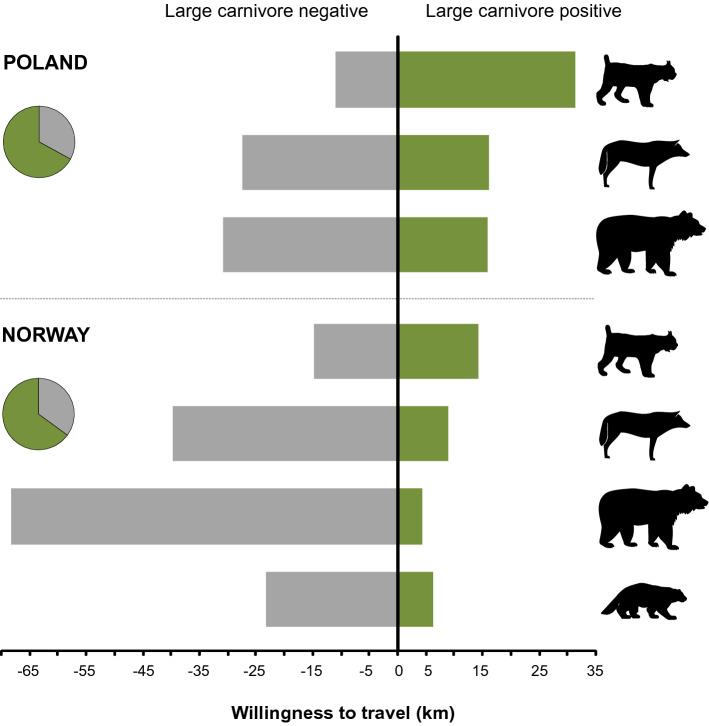
Estimates for the willingness-to-travel (kms) shown by respondents belonging to the large carnivore positive (green) and large carnivore negative (grey) classes in Poland and Norway according to the Latent Class Multinomial Logit model. Pie charts indicate the share of the two classes of respondents for each country.

In both countries, the most preferred LC species was the *Lynx*. Respondents were willing to travel an extra distance of 5.3 km in Norway and 11.4 km in Poland to visit a forest where *Lynx* were present (Table 3, Fig. 3). In the case of the *Bear* and the *Wolf,* the mean willingness-to-travel was negative in both countries, indicating that on average the presence of these two species decreased the recreational value of a forest. For the *Wolf*, the mean willingness-to-travel was − 5.0 km in Norway and − 5.2 km in Poland. In the case of the *Bear*, the respondents in Norway were willing to travel an extra 13.1 km to avoid visiting a forest with bears and in Poland 5.2 km (Table 3, Fig. 3). The *Wolverine* presence in the forests in Norway was perceived as neutral on average, as the mean estimate of willingness-to-travel for this species was not statistically different from 0.

Evaluating only the means of estimated distributions of willingness-to-travel may conceal the true preference patterns. We observed that, despite relatively low mean willingness-to-travel estimates (in absolute terms), the corresponding SDs of the estimated normal distributions of willingness-to-travel were very large and highly significant. This indicated a very large preference heterogeneity for the presence of LCs in the forest. The estimates we obtained implied that 59% of the respondents in Norway and 64% in Poland perceived *Lynx* presence as an element which positively contributed to forests’ recreational value (Table 3), and the remaining respondents, 41% in Norway and 36% in Poland, perceived *Lynx* presence in the forest negatively. The *Wolf* was perceived as contributing positively to the recreational value by 42% of the respondents in Norway and 44% in Poland. A very similar result was found for the *Wolverine*; 48% of Norwegians perceived the presence of *Wolverines* positively. The *Bear* was on average the least preferred species, its presence was perceived positively by 37% of the respondents in Norway and 43% in Poland.

### Linking preferences for LC presence and forest structural characteristics

The Latent Class Multinomial Logit model allows for the identification of latent classes of respondents with distinct preferences for forest attributes, including the presence of LC species. This model assumes that preferences are uniform within groups of individuals, but vary among these groups. We tested Latent Class models with two and three classes. The results of the model with three classes indicated that the preferences in both countries were highly polarized and that the proportion of people who were neutral towards the presence of LC in the forest was very small, 6% in Norway and 9% in Poland. Thus, to keep the results concise, we focused our attention on the model with two classes (Class 1 and Class 2; see below).

For both countries, the willingness-to-travel estimates in Class 1 for all LC species were negative and statistically significant, whereas the estimates in Class 2 for all species were positive and also statistically significant. Therefore, in both countries, we termed Class 1 as LC-negative and Class 2 as LC-positive. The probabilities of belonging to the LC-positive class were very similar in both countries (0.68 for Poland and 0.66 for Norway) and twice the probability of belonging to the LC-negative class (Table 4). Because most respondents in our study were assigned to the latent classes with a probability close to 1, class probability can be interpreted as the proportion of respondents in a given class. Thus, in both countries, two-thirds of the respondents perceived LCs as contributing positively to recreational value.

**Table 4:** Output of the Latent Class Multinomial Logit model assessing the public preference for forest attributes and large carnivore presence, expressed as willingness-to-travel, in Poland and Norway for the two classes of respondents (LC-positive and LC-negative).

Forest attributes	Poland				Norway			
	Class LC-negative		Class LC-positive		Class LC-negative		Class LC-positive	
	coef	t-stat	coef	t-stat	coef	t-stat	coef	t-stat
Broadleaved 1	1.01	0.44	− 9.76***	− 3.01	–	–	–	–
Broadleaved 3	8.70***	3.86	16.41***	5.85	–	–	–	–
Mixed 2	6.93***	3.21	10.95***	3.15	5.45**	2.41	10.88***	10.56
Mixed 4	10.57***	4.66	20.61***	8.56	–	–	–	–
Age-70	4.91**	2.36	27.76***	6.55	8.78**	3.04	6.28***	3.97
Age-100	1.85	0.67	38.36***	6.63	8.14**	2.51	10.43***	5.46
Two-aged	1.31	0.80	8.49***	5.07	− 7.65**	− 2.36	3.92***	2.88
Multi-aged	− 3.51*	− 1.87	9.72***	3.89	− 4.6	− 1.38	5.40***	4.09
Dead wood—Medium	− 1.66	− 0.94	3.35	1.15	0.36	0.14	4.84***	3.97
Dead wood—High	− 2.04	− 1.21	10.49***	5.63	− 7.21**	− 2.38	0.41	0.34
Bear	− 33.47***	− 10.60	15.09***	7.83	− 66.32***	− 6.97	4.27***	3.68
Lynx	− 11.14***	− 6.34	30.12***	11.92	− 14.59***	− 4.43	14.48***	13.05
Wolf	− 29.18***	− 10.15	15.19***	7.90	− 37.52***	− 6.37	8.74***	7.95
Wolverine	–	–	–	–	− 22.54***	− 5.22	6.39***	6.20
**Average class probabilities**								
	32.27***	19.53	67.73***	19.53	34.02***	35.72	65.98***	35.72
**Model diagnostics**								
Log-likelihood	− 9182.15				− 8259.95			
Pseudo-R^2^	0.1673				0.1641			
Observations	8776				8040			
Respondents	1097				1005			

The levels of significance are as follows: *0.1, **0.05, ***0.01.

The respondents in both countries and in both classes systematically preferred older forest stands. For example, respondents in the LC-positive class were willing to travel an extra 38 km in Poland and 10 km in Norway to visit a forest with *100-year-old* trees instead of a forest with *40-year-old* trees. The corresponding estimates in the LC-negative class for both countries were also positive for *Forest age*, but at a substantially smaller level, i.e., 1.9 km in Poland and 8.1 km in Norway for the *100-year-old *stand level. Apart from two forest structural characteristics (*Forest type* and *Age*), the remaining attributes were either not significant (*Age variation*, *Dead wood* for Poland, or the *Multi-aged* and *Medium level of dead wood* for Norway) or had negative willingness-to-travel values (*Two-aged* level of age variation or *High level of dead wood* in Norway).

For the LC-positive class, the importance of the presence of LC species in the forest was of similar magnitude to the most appreciated forest characteristics, e.g. willingness-to-travel of 28.6 for *100-year-old* and 21.1 km for *Mixed 4* stand in Poland, respectively, or 10.4 for *100-year-old* and 10.8 for *Mixed 2* in Norway (Table 4). In the LC-positive class, the highest willingness-to-travel was for the *Lynx* (14.5 km in Norway and 30.1 km in Poland), and the lowest was for the *Brown bear* (4.3 km in Norway and 15.1 km in Poland), with the *Wolf* being intermediate (8.7 km in Norway and 15.2 km in Poland, Table 4, Fig. 3). The willingness-to-travel for the *Wolverine* in Norway was 6.4 km.

Respondents in the LC-negative class in both countries had an unequivocally negative perception of LCs in the forest (Table 4, Fig. 3). They were willing to travel longer distances to avoid visiting forests in which any of the LC species were present. The negative preferences were more pronounced in Norway than in Poland. For example, Norwegian respondents in this class were willing to travel 66.3 km in order to avoid visiting a forest where *Brown bears* lived and 33.5 km to avoid visiting a forest with *Wolves*. In Poland, however, the perceptions of this class were less negative and willingness-to-travel estimates were -33.5 km and -29.1 km for the *Brown bear* and the *Wolf*, respectively. The least negatively perceived species in the LC-negative class was the *Lynx*, for which willingness-to-travel was -14.6 km in Norway and -11.1 km in Poland (Table 4, Fig. 3).

## Discussion

Our study clearly shows that LC species are important components of forest ecosystems also from a social perspective and that their presence significantly affects forest recreational value. In spite of the increasing interest on the socio-ecological aspects of human-carnivore relationships in the last decades, the available literature is biased and mainly focused on conflicts rather than on ecosystem services^29^. This pattern is particularly strong for European LCs, whose assessed impacts on the main three domains (economic, health and well-being, social and cultural) are predominantly negative, especially for wolf and bear studies, which deal mostly with negative economic impacts, such as damage to livestock^30^. Here, we showed that LCs can provide cultural ecosystem services that go beyond their simple existence and that their value for forest visitors, quantified as willingness-to-travel, was positive for most respondents. Reported non-material contributions of carnivores to people have referred mainly to recreational hunting and eco-tourism opportunities^29^, whereas most valuation studies applied to wildlife have focused on the recreational value of hunting^31^. In this sense, our study represents an original non-material contribution of LCs and helps to fill the research gap on the ecosystem services they provided^29,30^. To our knowledge, this is the first estimate of the recreational value of European LCs not related to hunting.

The positive recreational value of the presence of LC species in the forest was associated with a positive perception of natural forest structural attributes, such as old stands, high tree age variation, and presence of dead wood. This indicates a preference for natural forest ecosystems, where functioning ecological processes could be perceived and forest management was moderate; these findings are in line with previous research [see also^18,27^]. This suggests that most respondents have a holistic view of forest ecosystems, favouring natural and relatively unmanaged forests, which we could term ‘wildness positive’. Wildness is understood as a function of naturalness and lack of human control^3^. Although respondents belonging to the LC-negative class also preferred visiting forests of old age, their preference for mature stands was not as strong as in the wildness-positive class and they clearly avoided multi-age stands and dead wood, i.e. attributes associated with natural forests. This indicates a preference in this group for managed forests without LCs, which we termed as ‘wildness-negative’. These opposing views of wildness reflect the dualism of human-nature relationships that has been previously described as biophilia (love of nature;^32^)- and biophobia (fear of nature) or as ecocentrism (valuing nature for its own sake) and anthropocentrism (valuing nature because of material or physical benefits it can provide to humans;^33^). Kaltenborn and Bjerke^34^ found that the ecocentric environmental value orientation is significant and positively correlated with a preference for wildlands. They also showed that ecocentrism is linked to positive attitudes toward LCs, whereas anthropocentric views are associated with negative attitudes towards LCs^35^. Our results are consistent with these findings and in line with previous studies indicating that the presence of LCs may be associated with positive feelings, such as interest and joy, but can also evoke negative emotions, such as disgust, stress or fear [e.g.^36^]. These perceptions of wildness, thus, include a range of emotions from “paradise” to “hell” and have both proponents and opponents in the public^37,38^.

These polarized views towards wild nature is a common pattern in Europe. In Switzerland, half of the sampled population was in favour of wilderness and half unsupportive^37^, whereas in the Netherlands, more than 80% of the respondents preferred wild landscapes^39^. We found that two thirds of the respondents in both Poland and Norway were wildness positive, which agrees with the strong support for wild lands previously found in Norway^34^ and for natural-looking forests observed in Poland^18^. We found an even stronger polarization in the preferences towards LC presence in the forest. LCs and their presence were either liked, with willingness-to-travel values being of similar magnitude as that for the most attractive forest structural attributes, or they were highly disliked, with negative willingness-to-travel values being in absolute terms even 7 times more negative than those for the most attractive forest structural attributes in the case of Norway, or 3 times in Poland. Although these LC-negative groups were a minority in both countries (33% in Poland and 35% in Norway), their negative attitudes towards LC presence in the forest were much stronger than those with positive attitudes. The only exception was the lynx, for which the positive willingness-to-travel was twice as large as the negative willingness-to-travel in Poland, whereas for Norway the degree of positive and negative preferences was at the same level (in absolute terms). Norwegian respondents showed stronger negative views in relation to LC presence.

Preferences for forest attributes in both countries were heterogeneous. This heterogeneity was associated primarily with preferences in relation to LC presence in the forest, as their coefficients of variation (measured by the ratio of the SD to the mean) were much larger than the coefficients of variations for forest attributes. Interestingly, the mean willingness-to-travel values for the presence of LCs in the forest were small in absolute terms, compared to the willingness-to-travel values for the forest structural attributes. Moreover, for some carnivore species, for example wolves in Poland or wolverines in Norway, the estimates from the Multinomial Logit model were not statistically different from 0. This mixture of small mean willingness-to-travel estimates and very large SDs obtained in the Mixed Logit model, combined with the observations from focus groups, which indicated that people had rather unambiguous opinions and rarely were neutral towards LCs, suggested that positive and negative attitudes towards LCs may cancel each other out. The higher share of respondents with positive attitudes towards LCs, and the stronger negative than positive preferences, explain why, on average, the mean estimates of preferences for LC species in the Multinomial Logit and Mixed Logit models were close to zero. This, in turn, suggests that the true preference pattern may be concealed with the Multinomial Logit and Mixed Logit models, justifying our use of a Latent Class Multinomial Logit model with two classes. Although mixed logit models have become the state-of-the art tool for modelling data from choice experiments, when preferences are highly polarized, as in our study, the mean estimates are uninformative and may even lead to inaccurate conclusions because positive and negative attitudes cancel each other. Latent Class Multinomial Logit models represent a better tool to capture dualism in public preferences in such cases.

Forest visitor preferences varied strongly among LC species. On average, the presence of wolves was perceived negatively in Norway, but neutrally in Poland, whereas wolverines were perceived neutrally in Norway. Respondents in both countries perceived the lynx as a species that contributed positively to the recreational value of forest, whereas bears were perceived the most negatively. A survey conducted in Norway in 2000 found a similar pattern of more negative attitudes displayed towards wolves and bears than towards lynx and wolverines^25^. Forest visitors with negative perceptions of LCs would travel the largest distances to avoid forests with bears. This may be related to fear towards this species, which in essence is the only LC representing a potential danger for humans^40^. Fear plays a crucial role in the perceptions and attitudes towards LCs and in the willingness-to-pay for related conservation policies^36,41^. Other studies have shown, however, a less positive attitude towards wolves than towards bears in Europe, probably related to livestock damages^42,43^. Among the numerous factors affecting attitudes towards LCs, such as gender, age, social group or education [e.g.^25,42,43^], direct experience with LCs is particularly relevant, because experiences can shape future attitudes of forest visitors. In our study, people who visited the forest more frequently and whose main purpose was to observe nature were particularly linked with positive attitudes to nature. Although Eriksson et al.^44^ found that direct experiences with wolves and bears in Sweden reduced their public acceptance and policy support, wolf encounters were reported as positive experiences by most people in Germany, and encountering wolves in the wild increased the desire for closer proximity of respondents with wolves^26^. In both studies, LC populations were expanding in the respective countries, as generally reported for Europe^23^, suggesting that the number of encounters with LCs may potentially increase with time and that positive interactions, such as observing a LC or its tracks and signs, are important to improve human-LC coexistence^30,45^.

The available evidence shows conclusively that nature contributes to human well-being in a myriad of ways and that ecosystems provide culturally-mediated benefits, such as recreation, and positive effects on physical and mental health (see reviews in^45,46^). Research on wildness preferences, conducted primarily in urban environments, has shown that wild spaces are increasingly preferred and have positive impacts on people health and well-being, as well as on childhood development^15,47^. Wildness provides unique values in an increasingly urbanized world^14^. LCs can act as symbols or signifiers of wildness and visitors may perceive a landscape (even degraded) as wild and/or authentic as long as signifiers are present^48^. The needs for feelings of wildness, including unmanaged forests with LCs, will probably increase in a world that is becoming crowded and mechanized^49^.

In spite of recent rewilding initiatives, conservation strategies based upon letting ecosystems evolve without human control are still controversial in Europe, which is still far behind the United States in recognizing the value of wildness^49^. Our study indicates that restoring nature and rewilding projects have the potential to generate extra recreational benefits. Passive restoration of forests both enhance their recreational value and generate potential synergies with biodiversity conservation targets. This also raises issues about the material contributions of timber extraction and LC hunting compared to the nonmaterial contributions to people of relatively unmanaged forests inhabited by LCs. Negative perceptions of wildness, mostly driven by LC presence, represented a minority, but were stronger. The current increase of LC populations in Europe has intensified the debate on human-wildlife conflicts, which is becoming polarized around economic damages and risks to human safety^30^. Public preferences are complex and their full understanding is necessary to pave the way for effective conservation, e.g. for rewilding initiatives in Europe. Broadening the range of human well-being dimensions considered in conservation science and incorporating the intangible benefits of wildness into decision making is pivotal^45,46^ to fully understand human connections to ecosystems holding different attributes and to improve the way we manage ecosystems and their components.

## Methods

### Survey and choice experiment

We developed a questionnaire aimed at the general public in Poland and Norway via an iterative process involving both experts and laypersons. The questionnaire was adapted to Polish and Norwegian conditions in relation to the structure and type of forest and presence of LC species. A first draft of the questionnaire was prepared based on discussions with LC and forest experts and economists with experience in conducting stated preference surveys. The survey was carried out in 2016 as computer-aided web interviews by the same professional survey company in both Poland and Norway, which ensured that the implementation of the survey was identical in both countries. Both samples were representative with respect to *sex, age, municipality size, education*, and *region*, variables that characterised each respondent (see Table 2). The online implementation of both national questionnaires was hosted on a server owned by Kantar Millward Brown in Warsaw. This ensured consistency of data collection in Norway and Poland. The survey lives up to the ethical standards of the participating universities. Millward Brown SA operates in full compliance with the applicable law, the International Code of Marketing and Social Research Practice – ICC/Esomar and ISO 20,252 standard. Consent was obtained from all survey participants.

We used choice experiments to elicit preferences for LC presence within the attributes of forest type and structure (Table 1). Choice experiment is a survey-based valuation technique used to simultaneously value different characteristics of a good^50^. This technique is increasingly used to estimate people's willingness-to-pay for environmental attributes^51^, and involves asking individuals to state their choice over sets of hypothetical alternatives. Each alternative is described by several characteristics, referred to as attributes, including costs. The responses are used to determine whether preferences are significantly influenced by the attributes and their relative importance^50^.

The crucial part of our choice experiment was the identification of the complete range of forest attributes and their quantity on a management-intensity gradient from more to less natural forests. As forests also provide social values (see^52^ for an overview), we included forest attributes that are known to be relevant to public preferences for forest recreation, such as the age of the forest, tree size, or the number of tree species^18,27^. As the respondents in our study were not experts, considerable attention was devoted to the proper understanding of the forest attributes. Respondents were familiarized with the attributes through written descriptions and carefully selected photographs. In addition, we prepared 270 illustrations depicting different combinations of forest characteristics (Fig. 1). This was achieved by manipulating a set of hand-drawn, coloured tree diagrams from^53^. Using illustrations in the choice experiment component of the study allowed us to present the forest characteristics in an accessible manner. An example of a choice card combining all used attributes presented to respondents is shown in Fig. 1. The list of forest attributes used (*Distance to forest*, *Stand age*, *Variation in tree age*, *Forest type*, *Number of tree species*, *Dead wood*, *Large carnivore presence*) is explained in Table 1.

### Choice models, design and utility specification

The choice sets employed in our study were prepared using a Bayesian d-efficient design optimized for Multinomial Logit models^54^. The prior values were taken from a pilot study conducted on a sample of 100 respondents in both Poland and Norway. The designs for both countries were optimized independently. All forest attributes other than *Distance to forest* were dummy-coded (Table 1). The utility of the status quo alternative was given by a constant. The levels of *Forest type* and *Number of tree species* were combined at the estimation stage. For Poland, these two attributes were recoded into four dummy coded variables: *Broadleaved 1*, *Broadleaved 3*, *Mixed 2* and *Mixed 4*, whereas for Norway only the *Mixed 2* level was estimated. In both countries, *Coniferous 1* was used as the reference level and all other levels were estimated with respect to *Coniferous 1* (Table 1).

In a discrete choice experiment, respondents were asked to identify their preferred alternative among a given set of available alternatives. Our data analysis followed the Random Utility Model^55^, which assumes that the observed choice of an individual *n* is the one he/she expects to provide him/her with the highest utility. His/her utility function *U*_*ni*_ can be decomposed into a systematic part *V*_*ni*_ and a stochastic part $\varepsilon_{ni}$. The probability *P*_*ni*_ that the decision maker *n* chooses alternative *i* instead of another alternative *j* of the choice set is $P_{ni} = \Pr (V_{ni} + \varepsilon_{ni} > V_{nj} + \varepsilon_{nj} \forall j \ne i)$. If $\varepsilon_{ni}$ is assumed to be independently and identically distributed following an extreme value type I distribution^56^, this probability has a closed form multinomial logit expression. The limitation of the standard multinomial logit, which can represent only the systematic preference variation but not random preference variations, is relaxed by assuming a mixing distribution that is not degenerated at fixed parameters. In the case of the Mixed Logit model, all distributions, except for the *Distance to forest* attribute (our proxy of cost), were assumed to be normal. The distance coefficient was assumed to follow a log-normal distribution. This is equivalent to imposing the economic theory-driven restriction that the marginal utility of money is expected to be positive for all respondents^56^.

Because we observed a very high preference heterogeneity regarding LC presence in the forest (suggested by very large and highly significant standard deviations and means close to zero), we also employed a Latent Class Multinomial Logit model in addition to the Multinomial Logit and Mixed Logit models. A Latent Class Multinomial Logit Model allows for the identification of a number of latent classes of respondents with distinct preferences for forest attributes, including the presence of LC species. Unlike a standard Mixed Logit model, which allows for a continuous distribution of preference, Latent Class Multinomial Logit models assume that preferences are uniform within groups of individuals, but vary among these groups^56^. Inside the classes, the probability of choosing a given alternative is described in the same way as for a Multinomial Logit model. When estimating Latent Class models, we assumed two and three latent classes. The Latent Class model was also estimated considering that the membership of respondents to either class was probabilistically determined by respondent’s age, gender, number of forest visits and whether the purpose of the last forest visit was observing nature (Table S2).

All preference estimates for the models were expressed in willingness-to-pay space^56^. A positive estimate of willingness-to-travel can be interpreted directly as an extra distance that a respondent would be willing to travel to experience a forest with a given attribute level compared to a base level. Alternatively, if the reported willingness-to-travel estimate is negative, we interpreted it as an additional distance that the respondent would be willing to travel to avoid visiting a forest with a given attribute level with respect to the base level. We reported the mean and standard deviation (SD) of the willingness-to-travel and of the non-distance parameters, as well the share of respondents with negative willingness-to-travel for a given attribute.

## Supplementary Information

Supplementary Information 1.

Supplementary Information 2.

## Data Availability

All data are available as Dataset S1.
