# Supplementary material for: Large carnivores and naturalness affect forest recreational value
Source: Sci Rep. 2022 Aug 11;12:13692. doi: 10.1038/s41598-022-17862-0 (PMC9372138; doi:10.1038/s41598-022-17862-0)
Supplement: Supplementary file 1 — Supplementary Information 1. [file 41598_2022_17862_MOESM1_ESM.docx]

Supplementary material for:

**Large carnivores and naturalness affect forest recreational value**

Marek Giergiczny^1δ^, Jon E. Swenson^2^, Andreas Zedrosser^3,4^, Nuria Selva^5δ*^

^1^Faculty of Economic Science, University of Warsaw, ul Długa 44/50 00-241, Warszawa, Poland

^2^Faculty of Environmental Sciences and Natural Resource Management, Norwegian University of Life Sciences, Box 5003, NO-1432 Ås, Norway

^3^Department of Natural Sciences and Environmental Health, University of South-Eastern Norway, N-3800 Bø in Telemark, Norway

^4^Institute for Wildlife Biology and Game Management, University for Natural Resources and Life Sciences, A-1180 Vienna, Austria

^5^Institute of Nature Conservation Polish Academy of Sciences, 31-120 Kraków, Poland

δ These authors contributed equally to this work

*Corresponding author, e-mail: nuriselva@gmail.com

This file includes:

**Table S1.** Descriptive data for Poland and Norway on country surface, human density, large carnivore populations and forest cover types.

**Table S2.** Output of the Latent Class Multinomial Logit model assessing the public preference for forest attributes and large carnivore presence, expressed as willingness-to-travel, in Poland and Norway for the two classes of respondents (LC-positive and LC-negative) in relation to social variables.

**Table S1.** Descriptive data for Poland and Norway on country surface (km^2^), human density, large carnivore populations and forest cover types. Data on country surface, total human population, human population density (inhabitants/km^2^ land) and country forest cover (%) were taken from the World Bank Open Data (<https://data.worldbank.org/>; accessed 22 June 2022). Data on large carnivore abundance in Poland was taken from the EIONET Portal- assessment of species conservation status in Poland under the Art 17 of the Habitats Directive (<https://nature-art17.eionet.europa.eu/article17/species/report/>, period 2013-2018) and, for Norwegian large carnivores, from the Norwegian Large Predator Monitoring Program (ROVDATA, <https://rovdata.no>, estimates on 09 June 2022). The share of coniferous forests within the country forested areas was extracted from the Statistical Yearbook of Forestry (2021) for Poland (available at <https://www.bdl.lasy.gov.pl/portal/Media/Default/Publikacje/GUS_lesnictwo_2021.pdf>) and from the National Forest Inventory (2011-2015) for Norway (available at <https://www.ssb.no/en/jord-skog-jakt-og-fiskeri/statistikker/lst/aar/2016-08-26>).

| **Data** | **Poland** | **Norway** |
| --- | --- | --- |
| Surface (km^2^) | 312,690 | 625,222 |
| Human population (millions) | 38.0 | 5.4 |
| Human density (people/km^2^) | 124 | 15 |
| Brown bear | 99-166 | 160 |
| Lynx | 123 | 284-403 |
| Wolf | 1190-1886 | 125-129 |
| Wolverine | - | 358-418 |
| Forest cover (% country) | 31.0 | 33.4 |
| Coniferous forest share (% forest cover) | 68.3 | 75.0 |

**Table S2.** Output of the Latent Class Multinomial Logit model assessing the public preference for forest attributes and large carnivore presence, expressed as willingness-to-travel, in Poland and Norway for the two classes of respondents (LC-positive and LC-negative). Membership of respondents in either class is probabilistically determined by respondent’s age (Age), gender (Men=1), number of forest visits in the last 12 months (Visits) and whether the purpose of the last forest visit was observing nature (Nature=1) (“Explanatory variables of class probability”). The levels of significance are as follows: * 0.1, ** 0.05, *** 0.01.

|  | **Poland** | | | | **Norway** | | | |
| --- | --- | --- | --- | --- | --- | --- | --- | --- |
|  | **Class LC-negative** | | **Class LC-positive** | | **Class LC-negative** | | **Class LC-positive** | |
| **Forest attributes** | **coef.** | **t-stat** | **coef.** | **t-stat** | **coef.** | **t-stat** | **coef.** | **t-stat** |
| Broadleaved 1 | 0.58 | 0.26 | -9.73*** | -2.98 | - | - | - | - |
| Broadleaved 3 | 8.01*** | 3.61 | 17.09*** | 5.98 | - | - | - | - |
| Mixed 2 | 6.60*** | 3.10 | 11.19*** | 3.17 | 5.25** | 2.27 | 10.85*** | 10.64 |
| Mixed 4 | 10.08*** | 4.52 | 20.84*** | 8.54 | - | - | - | - |
| Age- 70 | 4.90** | 2.39 | 28.42*** | 6.56 | 8.75*** | 2.96 | 6.32*** | 4.05 |
| Age- 100 | 1.99 | 0.73 | 39.09*** | 6.64 | 8.03** | 2.40 | 10.37*** | 5.52 |
| Two-aged | 1.59 | 0.99 | 8.45*** | 5.00 | -7.76** | -2.34 | 3.86*** | 2.86 |
| Multi-aged | -3.16* | -1.72 | 9.90*** | 3.91 | -4.44 | -1.31 | 5.26*** | 4.02 |
| DW- Medium | -1.71 | -0.98 | 3.45 | 1.17 | 0.49 | 0.19 | 4.74*** | 3.95 |
| DW - High | -2.05 | -1.24 | 10.86*** | 5.75 | -7.26** | -2.34 | 0.34 | 0.29 |
| Bear | -32.32*** | -10.67 | 15.49*** | 7.82 | -67.89*** | -6.98 | 4.02*** | 3.62 |
| Lynx | -11.08*** | -6.44 | 30.60*** | 11.77 | -14.98*** | -4.43 | 14.35*** | 13.17 |
| Wolf | -28.32*** | -10.15 | 15.36*** | 7.84 | -39.19*** | -6.52 | 8.64*** | 7.93 |
| Wolverine | - | - | - | - | -23.31*** | -5.23 | 6.26*** | 6.20 |
|  | **Explanatory variables of class probability** | | | | | | | |
| **Social variables** |  |  | **coef.** | **t-stat** |  |  | **coef.** | **t-stat** |
| Age |  |  | -0.3064***  0.3064*** | -3.84 |  |  | -0.2612*** | -3.25 |
| Men |  |  | 0.2917*** | 4.06 |  |  | 0.4059*** | 5.06 |
| No. forest visits |  |  | 0.2198*** | 3.08 |  |  | 0.2582*** | 3.58 |
| Visit to nature |  |  | 0.3235*** | 4.52 |  |  | 0.2837*** | 3.79 |
|  | **Average class probabilities** | | | | | | | |
|  | coef. | t-stat | coef. | t-stat | coef. | t-stat | coef. | t-stat |
|  | 32.8432*** | 1.6241 | 67.1568 | 1.6241 | 33.5854*** | 1.7187 | 66.4145*** | 1.7187 |
|  | **Model diagnostics** | | | | | | | |
| Log-likelihood convergence | -9146.95  0.1705  8776  1097 | | | | -8226.68 | | | |
| Pseudo-R² | 0.1705 | | | | 0.1675 | | | |
| *Observations* | 8776 | | | | 8040 | | | |
| *Respondents* | 1097 | | | | 1005 | | | |
